# Successful therapeutic approach in a patient with elephantiasic pretibial myxedema^☆^^☆☆^

**DOI:** 10.1016/j.abd.2019.02.007

**Published:** 2019-11-25

**Authors:** Marina Ferreira, Luciana Helena Zacaron, Annair Freitas do Valle, Aloisio Carlos Couri Gamonal

**Affiliations:** a University Hospital, Universidade Federal de Juiz de Fora, Juiz de Fora, MG, Brazil; b Graduate and Post-Graduate Program in Dermatology, Faculdade Suprema, Juiz de Fora, MG, Brazil

**Keywords:** Evaluation of results of therapeutic interventions, Myxedema, Steroids, Therapeutics

## Abstract

Localized pretibial myxedema is a dermopathy whose treatment is a challenge in dermatology, occurring in 0.5–4% of patients with Graves’ disease. This autoimmune thyroid condition stimulates the production of hyaluronic acid and glycosaminoglycans that are deposited particularly in the pretibial region. Clinically, it presents as a localized, circumscribed, and non-depressible infiltrate in plaques. Several treatment modalities have been proposed, and their results vary, with worse response observed in severe cases. This report presents the case of a patient with elephantiasic pretibial myxedema who was subjected to intralesional corticosteroid applications, resulting in an excellent and encouraging therapeutic response that was maintained.

## Introduction

Localized pretibial myxedema is an infrequent manifestation of autoimmune thyroid diseases, especially Graves’ disease.^1^, ^2^ Its prevalence varies between 0.5% and 4% and it is more frequent in those with severe ophthalmopathy.^3^ Commonly, it appears as localized infiltrated plaques, circumscribing the pretibial region.^4^ Its management is challenging in dermatology.^2^ Several treatments have been proposed, ranging from compression stockings to intravenous immunoglobulin, generating a mild to moderate response, with unpleasant results in severe cases.^1^, ^4^ The current article describes the authors’ experience with intralesional corticotherapy in patient who present with the elephantiasic form, noting a satisfactory and encouraging clinical response during the follow-up of over 11 months.

## Case report

A 47-year-old female patient has had Graves’ disease since 2005. Four years ago, she presented with elephantiasic-like myxedema on the lower right limb, with a similar condition, to a lesser extent, on the lower left limb one year ago. The patient developed bilateral exophthalmos and severe retro-orbital impairment. She was subjected to orbital decompression and iodine therapy in 2012, developing hypothyroidism while maintaining clinical stability with levothyroxine use.

**Figure 1:**
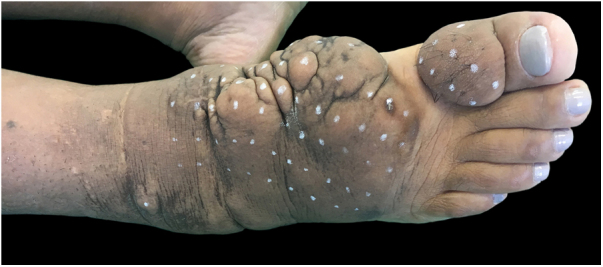
Clinical aspect of the right foot and ankle: confluent brownish nodules on waxy plaque. Hypertrichosis and hyperpigmentation are noted. First session of intralesional application of corticosteroid.

**Figure 2:**
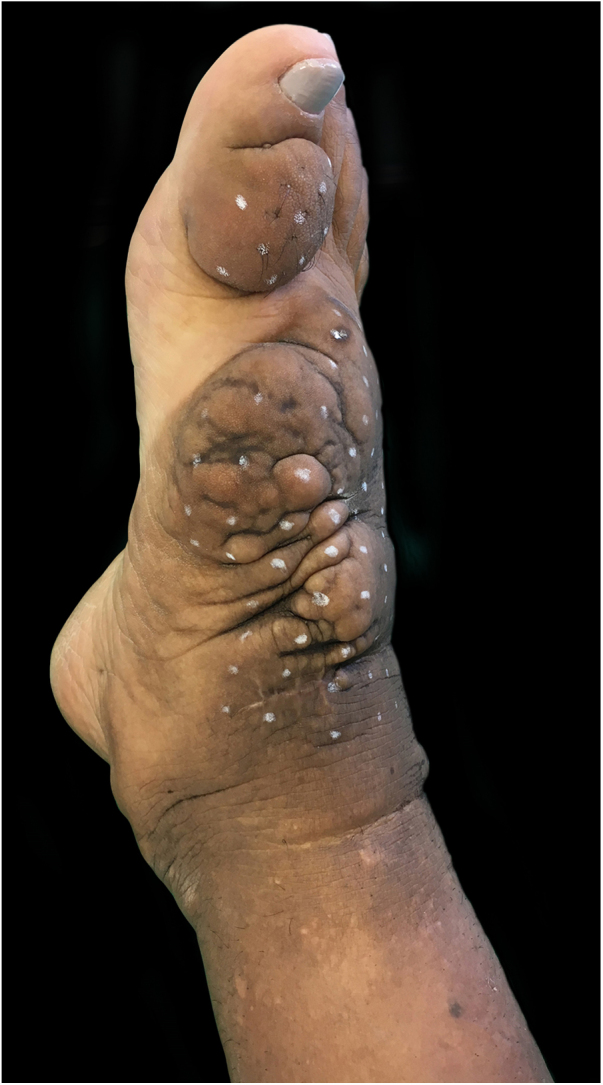
Clinical appearance of the right foot and ankle at the first session of intralesional application of corticosteroid. Non-depressible edema associated with hyperpigmentation reaching the lower third of the right limb, including lateral and posterior portions.

On clinical examination, she presented with non-depressible edema, associated with nodules and yellowish-brown plaques that formed an elephantiasiform pattern on the lower right limb, on the ankle and foot regions, in addition to hyperpigmentation and fissures with hypertrichosis on the dorsum of the foot and phalanges, and to a lesser extent, non-depressible edema in the lower left limb (LLL) (Figure 1, Figure 2).

**Figure 3:**
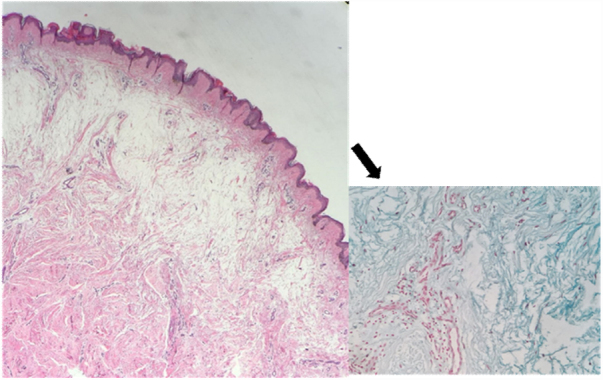
Important spacing of the collagen bundles of the upper reticular dermis, slight increase in the number of fibroblasts. Abundant deposit of mucin (arrow) (Hematoxylin & eosin, ×200) (Alcian blue, ×400).

The histopathological examination showed hyperortokeratosis in the epidermis, an intense deposit of mucin between collagen bundles in the reticular dermis, viewed in red on alcian blue coloration, that was compatible with myxedema cutaneous (Fig. 3).

In light of confirmation of the clinical condition and the patient's desire to improve her appearance, a therapeutic program was established with the use of triamcinolone acetate 20 mg/mL without dilution, applied over 50 points, 0.1 mL per point deposited through a 26G 1/2 needle into the reticular dermis, with the distance between two application points standardized at 1.0 cm. The following areas were treated: dorsum and lateral region of the right foot, proximal phalanx of the right first toe, and the left ankle. The initial frequency of the procedure was monthly. After four months and based on a very satisfactory clinical response, the interval between applications was increased to bimonthly, with a reduction of the compound that was administered by 50%, maintaining a satisfactory clinical response.

**Figure 4:**
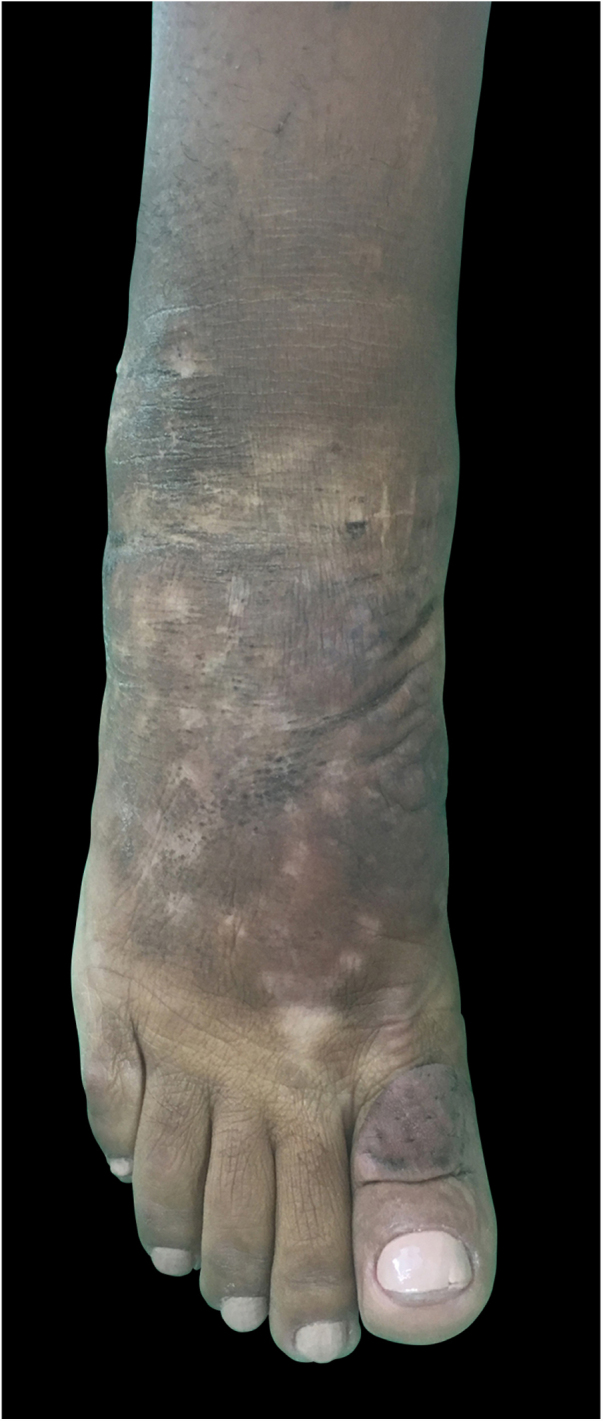
Clinical appearance of the right foot and ankle after 11 months of intralesional corticosteroid therapy. Almost total reduction of nodules and edema, making the appearance resemble the contralateral foot.

**Figure 5:**
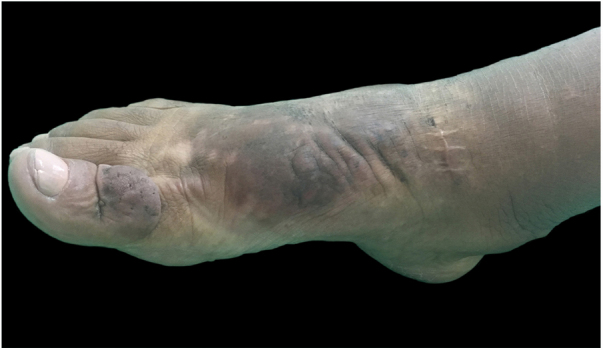
Clinical appearance of the right foot and ankle after 11 months of treatment with intralesional corticosteroid therapy. An important reduction of nodules and cutaneous texture is observed.

A clear decrease of non-depressible edema and nodules was observed, improving the coloration of the affected skin, allowing the patient to wear shoes that she was previously unable to due to her condition (Figure 4, Figure 5).

## Discussion

Graves’ disease is triggered by the emergence of antibodies against TSH receptors. Exophthalmia, acropathia, and pretibial myxedema are associated conditions and represent late manifestations, affecting 15–50 per 100,000 people per year, primarily women. Exophthalmia is usually present in patients with myxedema, in approximately 15% of cases.^3^, ^4^, ^5^

The etiology of myxedema is unknown, but it is speculated that stimulation of anti-TSH receptor leads to the proliferation of fibroblasts, causing an increase in hyaluronic acid and glycosaminoglycans and the consequent accumulation of fluids and compression of the small local lymphatics. Other proposed causal factors are venous stasis and local trauma.^4^

Clinically, the lesions are light-colored, but may also appear as yellowish-brown to reddish-brown. Hyperpigmentation, hyperkeratosis, fissures, hiperhidrosis and hypertrichosis are also present. Lesions may have a dense aspect, with the prominence of hair follicles, generating the *peau d’orange* sign. This dermopathy may be classified into four types: non-depressible edema that is accompanied by changes in color, plaques, nodules, and elephantiasiform.^1^, ^5^ The most commonly affected location is the anterolateral region of the lower limb extremities, possibly extending to the posterior face and feet. The condition may persist for months to years, and there are rare cases of spontaneous regression, especially in localized conditions. Elephantiasic forms are rare, typically progressive and refractory to treatments, leading to severe functional and emotional damage,^6^ as presented in this report.

Treatment with topical medium- to high-potency corticosteroids under occlusion has been described, generating a favorable clinical response but with a higher failure rate with regard to long-term remission.^1^, ^4^ Trials with intralesional triamcinolone have reported therapeutic success and a higher percentage of complete remission in three to four years, including dramatic responses, without the recurrence of lesions, as observed by Kumaran et al. in 2015.^4^ Several studies have combined medications, such as pentoxifylline and topical or oral corticosteroid, with less effective results in the cases of severe forms (elephantiasiform and diffuse).^4^

In 2015, Lan et al. compared the use of intralesional triamcinolone acetate in patients with several clinical forms of localized myxedema, reporting satisfactory responses in all presentations, corroborating the therapeutic initiative and the technique performed in the patient.^2^

The injection technique with corticosteroids, due to their anti-inflammatory and immunosuppressive properties,^7^ generates a significant and permanent reduction in pretibial dermal infiltrates in these patients, without causing degeneration, atrophy, or hyperpigmentation after application. In addition, it is possible to maintain these results,as in the case of the patient after 11 months.

Thus, the present patient's therapeutic success using only intralesional corticosteroid without other adjuvant treatments was associated with a rapid clinical response and the absence of systemic side effects, producing a positive emotional and social impact and encouraging the use of this technique in other patients with similar cases.

## Financial support

None declared.

## Authors’ contributions

Marina Ferreira: Composition of the manuscript; intellectual participation in the propaedeutic and/or therapeutic conduct in the studied cases; critical review of the literature.

Luciana Helena Zacaron: Approval of the final version of the manuscript; intellectual participation in the propaedeutic and/or therapeutic conduct in the studied cases; critical review of the literature; critical review of the manuscript.

Annair Freitas do Valle: Approval of the final version of the manuscript; intellectual participation in the propaedeutic and/or therapeutic conduct in the studied cases; critical review of the literature; critical review of the manuscript.

Aloisio Carlos Couri Gamonal: approval of the final version of the manuscript; conception and planning of the study; critical review of the literature; critical review of the manuscript.

## Conflicts of interest

None declared.
